# Benefits of STRENOLD Program on Health-Related Quality of Life in Adults Aged 60 Years or Older. In Common Sport Study

**DOI:** 10.3390/ijerph18063253

**Published:** 2021-03-21

**Authors:** Irimia Mollinedo-Cardalda, Adriana López Rodríguez, Manuela Ferreira, José María Cancela-Carral

**Affiliations:** 1 Faculty of Physiotherapy, University of Vigo, 36005 Pontevedra, Spain; 2 Faculty of Education and Sports Science, University of Vigo, 36005 Pontevedra, Spain; adrianalpez102@gmail.com; 3 Camara Municipal of Vilanova da Cerveira, 4920 Vilanova de Cerveira, Portugal; manuelaferreira11@hotmail.com; 4 Galicia Sur Health Research Institute (IIS Galicia Sur), Sergas-UVIGO, HealthyFit Research Group, Faculty of Education and Sports Science, University of Vigo, 36005 Pontevedra, Spain; chemacc@uvigo.es

**Keywords:** physical exercise, older adults, quality of life, strength, training

## Abstract

Background: The proportion of older adults is increasing worldwide and, with it, the physical inactivity common to this age group. Therefore, the promotion of active aging is a strategic factor in health policies for older people. The aim of this study was to identify the benefits and viability of the strength training program (STRENOLD) in health-related quality of life in adults over 60. Methods: A controlled experimental study was carried out with a sample of 181 people over 60 years old from different European countries belonging to the European project IN COMMON SPORTS. A pair work strength program was administered (STRENOLD) over a period of 24 months, consisting of two single sessions per week. Their health status was evaluated (EQ-5D-5L) before and after the interventions. Results: The adherence rate was over 89% and the tolerability rate over 100% in all participating countries. Significant improvements in the participants’ health were demonstrated in the areas of mobility, usual activities, pain/discomfort, and anxiety/depression. Conclusions: The regular practice of physical exercise, through the partnered STRENOLD strength program, has benefits on mobility, usual activities, pain/discomfort, and anxiety/depression, in short, health benefits for older adults.

## 1. Introduction

The proportion of older adults is increasing rapidly worldwide, and the proportion of people over 65 is projected to rise from 8% to 16% by 2050 [1]. Human aging causes a decline in physiological functions and physical capabilities, including: muscle mass, agility, flexibility, stamina, balance, strength, respiratory capacity, and bone mass [2]. The decrease in strength, along with other factors, such as the aging of the somatosensory and motor nervous system, produces functional implications such as decreased functional autonomy and impaired mobility (difficulty walking, increased risk of falling) as well as a reduced ability to perform normal daily activities [3]. All this contributes to the loss of independence of older people, diminishing their quality of life [4].

Physical inactivity among older adults increases progressively with age despite the multiple health benefits of regular and systematized physical activity [5]. The proportion of older adults who meet the physical activity guidelines recommended by the World Health Organization [1] declines with age and varies from 30% to 60% in people over 60 worldwide. In Europe, 35% of adults are considered physically inactive and this proportion increases with age to 45% of those over 60 [6]. Recently, a worldwide survey showed that the overall age-standardized prevalence of insufficient physical activity is 27.5%, and in Europe, the prevalence is 23.4% [7]. This prevalence of physical activity is different in European countries, people in southern Europe being more active than people in central and northern Europe, due to such various factors as climate, topography, and culture [8].

These low levels of physical activity can compromise functional capacity during the aging process [9]. Therefore, promoting active aging is a strategic factor in a health care policy for older adults. It has been demonstrated that older people who are physically active have a better quality of life, improved functionality, and better balance [10,11]. Of all the different options of physical activity that can be carried out by older people, strength training seems to occupy a prominent role since it directly influences both the functionality and the quality of life of older people [12] and is a determining factor in maintaining the independence of older adults [13].

Borba et al. [14] have stated that strength training is the most beneficial option since its effects on functional independence and the biological aging process itself lead to a decrease in the risk of falls and fractures and also to improvements in people’s quality of life. Bacelar et al. [15]; Poblete, Flores, Abad, and Diaz [16]; Marcos-Pardo et al. [17]; and Mora and Valencia [18] showed that strength training improves not only older adults’ quality of life but also their bodily composition at a skeleto-muscular level, increasing functionality and strength levels and giving the older adult more autonomy and independence. Dantas and Vale [19] showed that people who are physically active tend to prolong their functional independence and quality of life, and therefore, this activity has an important role in healthy aging. For these reasons, physical activity in general and strength training in particular lead to improvements in both health and the quality of life for older people [20,21].

For these reasons, the objective of the present study was to identify which benefits can be brought to the health-related quality of life in European adults over 60 years old by undertaking the strength training program (STRENOLD).

## 2. Materials and Methods

### 2.1. Participants

A sample of 181 participants (79.4% women) with an average age of 69.24(6.94) was recruited for the European Erasmus+ “IN COMMON SPORTS” project, in which four European countries participated: Bulgaria, Hungary, Italy, and Spain. This project promoted physical activity in 60+ year old residents of Europe, in line with the ethical principles of the Declaration of Helsinki. Table 1 shows the sample characteristics by countries. It should be noted that the groups are not homogenous in terms of gender, academic level, and pathologies. The inclusion criteria employed were the same as those used when implementing the European project: (1) Age 60+ years, (2) residents in the city where the program was implemented or in its vicinity, (3) having a medical certificate that allows participation in the “IN COMMON SPORT” training program, (4) ability to walk continuously for a minimum of 6 min, and (5) not playing competitive sports. The ethical standards contained in the Declaration of Helsinki were followed in this study, and this study was approved by the Polytechnic Institute of Viana do Castelo Ethical Committee (IPVC-ESDL180417). In addition, all participants signed an informed consent prior to their participation in the program. The “IN COMMON SPORTS” program recruited participants voluntarily through the websites and press advertisements that each municipality publicized, where the project and the sign-up procedure were announced.

### 2.2. Measurements

#### 2.2.1. Feasibility

The following data were gathered to evaluate feasibility: recruitment rate (number of participants recruited vs. number of participants who met the inclusion criteria); participation rate (total completed hours of exercise vs. total possible hours of exercise); adherence (rate of patients with 80% participation or higher); dropout (number of participants who could not complete the program); safety and tolerability (number of patients who suffered adverse effects derived from the intervention, such as pain, dizziness, vertigo, falls, etc.).

#### 2.2.2. Health State

The 5-level EQ-5D version (EQ-5D-5L) was introduced by the EuroQol Group in 2009. It is a simple, generic, and standardized instrument for assessing the state of patients’ health. [22]. The EQ-5D-5L is widely used with all types of populations and ages [23]. This test consists of two parts: the EQ-5D descriptive system and the EQ visual analog scale (EQ VAS). The descriptive system of the states of quality of life related to health in adults has five dimensions: mobility (MO), self-care (SC), usual activities (UA), pain/discomfort (PD), and anxiety/depression (AD). Each dimension has five levels (no problems, mild problems, moderate problems, severe problems, and extreme problems), where level one means no problems and five means having severe and extreme problems. This part of the EQ-5D questionnaire provides a descriptive profile that can be used to generate a health status profile. An example of this health status profile is the code 12231, which means that the person does not have mobility problems or depression, but also does not have mild self-care problems or usual activities, and moderate problems with pain/discomfort. The second part of the questionnaire consists of a visual analog scale (VAS) in which the patients rate their perceived health from 0 (the worst imaginable health) to 100 (the best imaginable health).

### 2.3. Intervention

The STRENOLD Program is a training program designed specifically for older people. The main objective is strength training combined indirectly with other conditional abilities (balance, stamina, coordination). What is relevant about this program is that it is carried out in pairs, using very little equipment (only a fitball and elastic bands) and maximizing the use of the bodyweight of the individual and partner. It is also important to highlight the social element of the pair work.

The STRENOLD Program is divided into three parts:

- Warm up (10 min), comprised of: cardio exercises (6 min) and joint mobility (4 min).
- Main part (45 min), comprised of: coordination exercises and aerobic games (10 min), strength exercises based on bodyweight and resistance (in pairs—15 min), balance (5 min), and Pilates/yoga (15 min).
- Cool down (5 min), comprised of: stretching (3 min) and relaxation (2 min) exercises.

The STRENOLD program was carried out over 24 months with two 60-min sessions per week by graduates in physical activity and sports sciences specialized in training older people. It is worth noting that, prior to the intervention, a meeting of specialists was held to agree on the program and the types of exercises to be implemented, as well as the different adaptations to be used according to the physical condition and functionality of the participants. The training in pairs was carried out after having considered their bodyweight and the functional level of each component.

### 2.4. Procedure

The representatives of the European project “IN COMMON SPORTS” contacted the participants in each member country to make a first assessment (baseline) of the individuals in March 2018. Within a week, all subjects were summoned simultaneously in all the participating countries (Italy, Spain, Bulgaria, Hungary). The assessments were made in sports centers (gyms, sports halls) where the subjects carried out their training. After the first evaluation, the STRENOLD program was carried out over a period of 24 months with two 60-min sessions per week. At the end of the STRENOLD program, a final assessment was carried out simultaneously in all the participating countries, and the data were dumped into the common SPSS statistical system, where the different statistical analyses were performed. In the statistical analysis, the sample was divided into groups comprising the four countries.

### 2.5. Statistical Analysis

A descriptive analysis was carried out through measurements of central tendency (mean and standard deviation) and percentages. The analysis was carried out globally, and also stratified by country, with the aim of defining the basic characteristics of the sample (age, sex, academic level, and main pathologies). We compared the basic characteristics of the sample by country to identify significant differences between countries using the one-way analysis of variance (quantitative variable) and chi-square test (qualitative variable). To analyze the feasibility of the STRENOLD program, the following parameters were calculated using percentages: recruitment rate, participation rate, adherence, dropout, safety, and tolerability. The state of the sample’s health was described by means of percentages and an average of the index and the visual analogue scale (VAS). In order to understand the effect of the STRENOLD program on the sample, an analysis was carried out using the paired Student’s t-test (quantitative variable) or chi-square test (qualitative variable). This analysis was carried out globally and stratified by country across the sample. Effect size (ES) was calculated for each comparison using Cohen’s d to evaluate the size of mean differences. The Bonferroni correction for multiple comparisons was applied. The normality of the variables within the study was verified using the Kolmogorov–Smirnov test (*p* > 0.05). The statistical analyses were carried out using the statistical package IBM-SPSS v25 for Mac. The significance was considered as *p* < 0.05.

## 3. Results

A total of 181 people over 60 years old from different European countries were selected to participate in the training sessions of the European IN COMMON SPORTS project. There was an overall recruitment rate of 64.25%, with Hungary having the highest rate at 94.44% (*p* = 0.01). The participation rate was 90.07%, showing an adherence of 91.14%. The drop-out rate was 3.09%, with Bulgaria (6.33%) and Italy (4.16%) showing the highest rates in this field. The safety and tolerability rate was 100% since no adverse effects deriving from the program were reported. These data are shown in Table 2.

Table 3 presents the health profiles with the highest frequency among the participants through the 5-digit scale (11111 to 55555) before and after the intervention. It should be highlighted that, before the intervention, 28.50% of the sample did not present any health problems, while the cumulative percentage of those presenting one or two mild health problems was 53%. After the intervention, severe health problems disappeared, there was a decrease in moderate problems, while the numbers of those who presented some slight health problems (56.30%) or no problems at all (32.10%) increased. There was a similar situation with the mean difference EQ-VAS score. These results show a significant trend towards the disappearance of severe health problems with increases in some slight health problems (ES = 0.38) and no problems at all (ES = 0.19).

Table 4 shows the changes that occurred in the five parameters of the EQ-5D-5L health state after applying the STRENOLD Program. Significant improvements were observed in all parameters EQ-5D-5L (mobility, self-care, usual activities, pain/discomfort, and anxiety/depression) and the EQ-VAS score. If we stratify the sample by country, all showed significant improvements in anxiety/depression.

Hungary and Bulgaria recorded the largest effect sizes (ES = 0.39 and ES = 0.29) in this parameter, and these countries were also the only ones to show significant improvements in pain/discomfort (ES = 0.18 and ES = 0.39). In the mobility parameter, Hungary, Italy, and Spain showed significant improvements with Spain recording the largest effect size here (ES = 0.29). These three countries also demonstrated significant improvements for usual activities with similar effect sizes (ES = 0.17–0.19). In the self-care parameter, only Spain showed significant improvements. Thus, significant improvements were observed in four parameters for Spain and Hungary, in three for Italy, and in two for Bulgaria.

In terms of the index score, only Hungary and Spain showed significant improvements, while in the EQ-VAS score Hungary, Spain, and Bulgaria reflected significant improvements.

## 4. Discussion

The population of older people is growing significantly and, with it, the number of associated health problems. Age-related illnesses are of great concern because they result in a marked deterioration in people’s quality of life. Therefore, the practice of physical activity becomes an ally of older adults, as a significant relationship between the amount of physical activity practiced and the functional level and health of the elderly population has been demonstrated [24].

Strength training has a positive influence on the quality of life and functionality of the elderly and is the most beneficial form of physical exercise [12]. In fact, the STRENOLD Program, based on strength training, showed a tendency to improve all the fields of EQ-5D-5L, in mobility, self-care, usual activities, pain/discomfort, and anxiety/depression, although the effect sizes are small. Therefore, our results are in line with previously published articles in which it is shown how strength training programs generate health benefits in this group [12,25]. In addition, an improvement in the EQ-VAS score was reflected with the medium effect size; perhaps it is a consequence of the fact that 58% of the sample presented with hypertension, a pathology that evolves favorably with the practice of physical activity [26].

The results of this study show that the same strength training program applied in four countries (Hungary, Bulgaria, Italy, and Spain) generated different results across the parameters of health status (EQ-5D-5L). Hungary and Bulgaria presented significant improvements in the pain/discomfort parameter (ES = 0.39 and ES = 0.18), while Spain presented significant improvements in self-care (ES = 0.15). Three countries (Hungary, Italy, and Spain) showed significant improvements in the usual activities and mobility parameters. These results reflect that, despite the application of the same strength program, there are differences depending on the place of residence. This may be due to the fact that quality of life is multifactorial and, therefore, affected by physical exercise and lifestyle, social limitations (dependence, social discomfort, stimulation from others), physical limitations (comorbidities), accessibility issues (architectural and environmental barriers), and motivations and beliefs [27].

It is noteworthy that all the countries shared a significant improvement in the anxiety/depression parameter, and thus, it is evident that the practice of physical activity by older adults improves states of anxiety or depression [28]. These states are the factors that contribute most towards a negative perception of quality of life. Many older people suffer from depression or anxiety due to changes in their lives—whether they are changes in routine, social changes such as the loss of friends and family, or changes in their bodies, such as the loss of physical abilities or the appearance of new age-related illnesses [29]. All this affects people’s ability to carry out simple daily tasks, thus, generating negative effects on their quality of life [30]. That is why programs like the one in this study, which are not carried out individually but in pairs, are increasingly necessary, since, thanks to them, older people can socialize with each other while participating in exercise that reduces the anxiety and depression they may suffer.

When considering the significant improvements in the parameters of mobility and usual activities, the economic, health, and educational factors—and the reasons why they carry out physical activity—should be taken into account. The countries of Spain, Italy, and Hungary have an annual gross domestic product (GDP) and a per capita GDP higher than Bulgaria, a factor which affects education, health, and ultimately the welfare of their citizens [31].

In keeping with the findings of the study by Lubs et al. [32], it could be said that the countries with a higher level of education (more years spent in the educational system) are those that carry out more physical activity.

Additionally, the reasons why this age group engages in physical activity may also be an important factor in these significant improvements (mobility, pain/discomfort and usual activity). It has been shown that the most common reasons to do sport or physical activity are to improve health (54%) and to improve physical fitness (47%) [8].

This suggests that the STRENOLD program is very important for this population group because of the multiple benefits associated with doing it.

However, at present, there is an increase in physical inactivity among European older adults [8]. This can lead to multiple negative consequences, such as decreased functional capacity, chronic diseases, obesity, cognitive dysfunction, sarcopenia, altered balance and equilibrium, falls and fractures, osteoarthritis, and osteoporosis [33,34]. These data regarding physical inactivity have increased since the onset of the coronavirus disease 2019 (COVID-19) pandemic. Remaining totally inactive for 1–2 weeks during isolation decreases major health determinants such as muscle strength and cardiorespiratory fitness [35]. Therefore, it can be said that isolation favored sedentary behavior and physical inactivity in older people who could not go out to exercise, thus, increasing their risk of developing diseases or of previous pathologies deteriorating [36,37]. Consequently, the importance of teaching older adults physical activity programs that can be done at home is clear. This is the case with the STRENOLD program, which, due to the minimal equipment necessary, is a program that can be done at home and in the company of another person, either a family member or caregiver. In addition, this program has shown high levels of reliability and tolerability, so we can suggest that it is a valid tool for the practice of physical exercise, and especially at present, one that is able to be performed at home. It must be remembered that the STRENOLD program has had positive effects on the health of our elders with the disappearance of severe health problems, a decrease in moderate problems, and an increase only in mild health problems after the completion of the intervention, in line with the findings of several studies [38,39,40], where it is stated that physical activity decreases health problems, whether mental or physical. The most relevant limitations of this pre-experimental study are that (a) it did not include a control group, (b) the sample size was not calculated, and (c) it used convenient samples, limiting its generalizability. However, the study contributed to the existing literature as, to the authors’ knowledge, no research-based articles have been published using paired strength training in older adults to date. Future research should test the effectiveness of the STRENOLD program in more European countries.

## 5. Conclusions

The results suggest that engaging in regular physical activity using the STRENOLD strength program (in pairs and without equipment, over 24 months with two 60-min sessions per week) can, albeit in a small way, bring about health benefits inasmuch as the participants reported improved feelings of well-being and emotional state. The results suggest that older adults show improvements in mobility, in self-care, and in the execution of daily activities and report decreasing levels of pain/discomfort and anxiety/depression. However, these benefits may be conditioned by external factors because of the variability of results in the countries where the program was implemented.

## Figures and Tables

**Table 1:** Sample Characteristics by Countries.

	All Countries *n* = 181	Bulgaria *n* = 45	Hungary *n* = 34	Italy *n* = 48	Spain *n* = 54	Chi-Square/Anova
**Gender (%)**						
Female	79.40	84.20	78.20	77.70	77.50	Chi = 6.508 *p* = 0.012
Male	20.60	15.80	21.80	22.30	22.50	
**Age (years)**	69.24 (6.94)	70.14 (7.78)	66.42 (5.94)	69.17 (7.37)	71.21 (6.67)	F = 6.833 *p* = 0.001
**Level academic (%)**						
Primary school	40.80	-	-	38.70	42.90	Chi = 56.266 *p* = 0.001
High school	53.62	63.20	54.50	50.00	46.80	
University	25.60	36.80	45.50	9.70	10.30	
Doctorate	1.60	-	-	1.60	-	
**Main pathology (%)**						
Parkinson	2.00	-	-	-	2.00	Chi = 70.754 *p* = 0.001
Cognitive impairment	2.90	3.80	-	-	2.00	
Multiple sclerosis	-	-	-	-	-	
Hypertension	43.87	54.00	48.80	36.70	36.00	
Asthma	3.20	-	0.80	-	5.60	
Chronic obstructive pulmonary disease	4.82	3.20	5.50	6.00	4.60	
Low back pain	3.50	2.00	-	-	5.00	
Osteoporosis	13.80	14.40	12.20	-	14.80	
Diabetes	8.42	2.00	4.20	3.50	24.00	
Cancer	16.90	13.60	24.50	23.50	6.00	
Others	5.10	7.30	4.00	4.00	-	

**Table 2:** Feasibility of STRENOLD program.

	All Countries *n* = 181	Bulgaria *n* = 45	Hungary *n* = 34	Italy *n* = 48	Spain *n* = 54	Chi Square
Recruitment rate (%)	64.26	52.32	94.44	55.17	55.10	Chi = 18.46; *p* = 0.01
Participation rate (%)	90.07	91.65	82.25	95.67	90.74	Chi = 4.59; *p* = 0.23
Adherence (%)	91.14	89.67	91.15	90.10	93.67	Chi = 3.13; *p* = 0.43
Dropout (%)	3.09	6.33	0.00	4.16	1.85	Chi = 9.35; *p* = 0.02
Safety and tolerability (%)	100.00	100.00	100.00	100.00	100.00	-

**Table 3:** EQ-5D-5L health state.

EQ-5D-5L Health State					Baseline		Post Intervention		EQ-VAS Score		
MO	SC	UA	PD	AD	N	Cumulative %	*n*	Cumulative %	Mean Difference	Student’s t	Cohen’s d
1	1	1	1	1	96	28.50	108	32.10	1.76	t = 2.60 *p* = 0.01	d = 0.19
1	1	1	2	1	30	37.40	34	42.20	0.99	t = 1.03 *p* = 0.05	d = 0.10
1	1	1	1	2	19	43.00	11	45.50	2.08	t = 3.28 *p* = 0.01	d = 0.22
1	1	1	2	2	17	48.00	17	50.60	0.03	t = 0.57 *p* = 0.11	d = 0.01
2	1	1	2	1	17	53.00	19	56.30	3.65	t = 2.65 *p* = 0.01	d = 0.38
1	1	1	3	1	11	56.30	3	57.20	3.57	t = 2.55 *p* = 0.01	d = 0.54
1	1	1	2	3	7	58.40	2	57.80	1.28	t = 1.87 *p* = 0.02	d = 0.23
3	3	3	3	3	7	60.50	7	59.90	0.21	t = 0.77 *p* = 0.07	d = 0.02
2	1	1	1	1	6	62.30	5	61.40	1.64	t = 1.98 *p* = 0.01	d = 0.17
1	1	1	3	3	5	63.80	0	61.40	-	-	-
2	1	1	2	2	5	65.30	13	65.30	3.93	t = 2.11 *p* = 0.01	d = 0.41
2	1	1	3	2	5	66.80	0	65.30	-	-	-
2	1	2	2	2	4	68.00	8	67.70	2.75	t = 2.01 *p* = 0.01	d = 0.28
1	1	1	1	3	3	68.90	1	68.00	23.75	t = 3.87 *p* = 0.01	d = 0.55
1	1	2	2	1	3	69.80	5	69.50	3.25	t = 1.95 *p* = 0.01	d = 0.33
1	1	2	2	2	3	70.70	3	70.40	5	t = 2.88 *p* = 0.02	d = 0.51
2	1	2	3	1	3	71.60	1	70.70	7.5	t = 3.12 *p* = 0.01	d = 0.77
2	1	2	3	3	3	72.50	0	70.70	-	-	-
4	4	4	4	4	3	73.40	0	71.60	-	-	-

Obs: AD, anxiety/depression; ES, Effect Size; MO, mobility; PD, pain/discomfort; SC, self-care; UA, usual activities; VAS, visual analogic scale. The Bonferroni correction for multiple comparisons was applied.

**Table 4:** EQ-5D-5L health state. Effects of the STRENOLD Program by country.

EQ-5D-5L Health State								
		Mobility	Self-Care	Usual Activities	Pain-Discomfort	Anxiety Depression	Index Score	EQ-VAS Score
**All countries** ***n*** **= 181**	Baseline	1.65 (0.86)	1.24 (0.61)	1.43 (0.74)	1.94 (0.89)	1.67 (0.93)	0.82 (0.19)	74.18 (16.65)
	Post intervention	1.47 (0.78)	1.23 (0.60)	1.32 (0.64)	1.83 (0.79)	1.51 (0.74)	0.83 (0.17)	77.69 (17.37)
	Paired *t*-test	t = 1.76; *p* = 0.01	t = 0.93; *p* = 0.04	t = 2.01; *p* = 0.01	t = 1.91; *p* = 0.01	t = 2.44; *p* = 0.01	t = 0.19; *p* = 0.16	t = 1.19; *p* = 0.04
	ES: Cohen’s d	d = 0.22	d = 0.02	d = 0.16	d = 0.14	d = 0.19	d = 0.05	d = 0.31
**Bulgaria** ***n* = 45**	Baseline	2.08 (0.95)	1.71 (0.96)	2.07 (0.97)	2.43 (0.88)	2.18 (1.05)	0.67 (0.24)	65.12 (16.03)
	Post intervention	1.87 (0.81)	1.76 (0.85)	1.91 (0.82)	2.13 (0.64)	1.92 (0.73)	0.70 (0.18)	71.04 (14.13)
	Paired *t*-test	t = 0.89; *p* = 0.06	t = 0.32; *p* = 0.12	t = 1.01; *p* = 0.05	t = 2.98; *p* = 0.01	t = 3.05; *p* = 0.01	t = 1.05; *p* = 0.07	t = 1.93; *p* = 0.01
	ES: Cohen’s d	d = 0.26	d = 0.05	d = 0.18	d = 0.39	d = 0.29	d = 0.14	d = 0.39
**Hungary** ***n*** **= 34**	Baseline	1.44 (0.76)	1.05 (0.40)	1.13 (0.47)	1.47 (0.74)	1.22 (0.53)	0.91 (0.16)	81.78 (12.05)
	Post intervention	1.24 (0.64)	1.03 (0.16)	1.06 (0.23)	1.35 (0.59)	1.06 (0.23)	0.95 (0.08)	81.49 (18.02)
	Paired *t*-test	t = 2.05; *p* = 0.01	t = 1.11; *p* = 0.06	t = 1.56; *p* = 0.01	t = 1.87; *p* = 0.01	t = 2.45; *p* = 0.01	t = 0.21; *p* = 0.01	t = 0.67; *p* = 0.01
	ES: Cohen’s d	d = 0.28	d = 0.06	d = 0.19	d = 0.18	d = 0.39	d = 0.11	d = 0.19
**Italy** ***n* = 48**	Baseline	1.49 (0.88)	1.11 (0.41)	1.26 (0.60)	1.88 (0.77)	1.74 (0.90)	0.84 (0.15)	76.68 (15.35)
	Post intervention	1.37 (0.64)	1.06 (0.25)	1.16 (0.44)	1.89 (0.78)	1.58 (0.73)	0.85 (0.13)	80.48 (15.02)
	Paired *t*-test	t = 2.18; *p* = 0.01	t = 1.57; *p* = 0.05	t = 2.57; *p* = 0.01	t = 0.93; *p* = 0.05	t = 2.67; *p* = 0.02	t = 0.88; *p* = 0.32	t = 1.11; *p* = 0.19
	ES: Cohen’s d	d = 0.16	d = 0.14	d = 0.19	d = 0.02	d = 0.20	d = 0.07	d = 0.25
**Spain** ***n* = 54**	Baseline	1.62 (0.84)	1.12 (0.52)	1.26 (0.54)	1.98 (0.84)	1.55 (0.85)	0.84 (0.14)	73.15 (20.63)
	Post intervention	1.42 (0.65)	1.06 (0.21)	1.17 (0.52)	1.97 (0.75)	1.46 (0.76)	0.83 (0.13)	77.76 (18.54)
	Paired *t*-test	t = 2.33; *p* = 0.01	t = 1.45; *p* = 0.04	t = 2.47; *p* = 0.01	t = 0.87; *p* = 0.05	t = 2.31; *p* = 0.04	t = 2.01; *p* = 0.04	t = 2.01; *p* = 0.04
	ES: Cohen’s d	d = 0.27	d = 0.15	d = 0.17	d = 0.01	d = 0.12	d = 0.07	d = 0.23

Obs. VAS, visual analogic scale; ES, Effect Size. The Bonferroni correction for multiple comparisons was applied.

## Data Availability

http://www.olympics4all.eu/ (accessed on 25 October 2020).
